# Impact of Combined Botanical Functional Ingredients on the Canine Gut Microbiome

**DOI:** 10.3390/vetsci13090877

**Published:** 2026-08-27

**Authors:** Estefania Morua, Lourdes Criado-Mesas, Guillermo Chumaceiro, Maialen López Palma, Roberto Malinverni, Walter Sanseverino, Riccardo Aiese Cigliano, Laura Cuyas, Luis Matías-Hernández

**Affiliations:** 1 Biotech Tricopharming Research, 08013 Barcelona, Spain; em@tricopharming.com (E.M.);; 2 Sequentia Biotech, 08005 Barcelona, Spain

**Keywords:** canine gut microbiome, Medicinal and Aromatic Plants, *Aloe vera*, *Artemisia annua*, *Curcuma longa*, metagenomics, short-chain fatty acids, gut microbiome modulation, functional botanical ingredients

## Abstract

The canine gut microbiome plays a key role in digestive, immune, and metabolic health and is increasingly recognized as an important indicator of overall animal well-being. Medicinal and Aromatic Plants are emerging as a promising approach to support intestinal balance through modulation of the gut microbiome. This pilot study evaluated the effects of *Aloe vera*, *Artemisia annua*, and *Curcuma longa* on the gut microbiome of healthy dogs. The study included eighteen healthy dogs, divided into an Experimental group (*n* = 9) and a Control group (*n* = 9). Dogs in the Experimental group received a daily combination of the three plants for 30 days, while Control dogs received microcrystalline cellulose as a placebo. Fecal samples were collected on Day 30 and analyzed using shotgun metagenomic sequencing. Differential abundance analysis revealed an enrichment of taxa associated with short-chain fatty acid production in the Experimental group, including *Megasphaera elsdenii*, *Blautia*, *Butyricimonas*, *Bacteroides*, and *Phascolarctobacterium*, together with a reduction in opportunistic and dysbiosis-associated taxa, such as *Enterocloster bolteae*, *Anaerobiospirillum* spp., and *Stenotrophomonas maltophilia*. Functional profiling showed a reduction in pathways related to virulence-associated mechanisms and iron siderophore systems, alongside an increase in pathways associated with GABA and putrescine metabolism and serine endopeptidase activity. Overall, these findings suggest that supplementation with these functional botanical ingredients may promote a more balanced and functionally beneficial gut microbiome in healthy dogs.

## Simple Summary

The gut microbiome is closely related to overall health and well-being. Plant-based functional ingredients are gaining attention as a natural strategy to support intestinal health. In this pilot study, we investigated the effects of a daily combination of *Aloe vera*, *Artemisia annua*, and *Curcuma longa* in healthy dogs. Dogs in the Experimental Group which received this combination showed higher levels of several bacterial groups associated with the production of beneficial compounds that support intestinal health, together with lower levels of some microorganisms linked to intestinal imbalance or opportunistic behavior. Changes were also observed in microbial functions related to bacterial metabolism and potential virulence mechanisms. These preliminary findings suggest that supplementation with these botanical ingredients may help promote a more balanced and potentially beneficial gut microbiome in healthy dogs.

## 1. Introduction

The intestinal microbiome comprises a diverse community of microorganisms that inhabit the gastrointestinal tract. In companion animals, it plays a central role in several physiologic processes, including metabolism, immune modulation, maintenance of gut epithelial integrity, and neurobehavioral development [1]. In recent years, research on the canine microbiome has advanced rapidly, enabling the identification of core microbial genera, as well as taxa that may have been lost during domestication and others acquired through close cohabitation with humans [2].

Although significant advances have been made, several important knowledge gaps remain regarding the mechanisms that shape the composition, function, and host interactions of the canine gut microbiome. Much of the current understanding of the canine gut microbiome continues to be extrapolated from human microbiome research; however, this extrapolation may not fully capture species-specific characteristics. Recent metagenomic studies indicate that, despite functional and taxonomic similarities between dogs and humans, the canine gut microbiome contains distinct microbial lineages and host-adapted features, underscoring the need for dog-specific microbiome datasets and mechanistic studies [3,4].

Alterations in the intestinal microbiome, commonly referred to as dysbiosis, have been increasingly associated with a wide range of diseases in dogs. These include gastrointestinal disorders, such as chronic enteropathies and diarrhea, as well as extra-intestinal conditions, including obesity, metabolic disorders, allergic diseases, and immune-mediated disorders [1,5]. Dysbiosis is typically characterized by reduced microbial richness and diversity, loss of beneficial commensal taxa, and expansion of potentially pathogenic or pro-inflammatory microorganisms. These alterations may disrupt microbial metabolism, intestinal barrier function, and host-microbe interactions, contributing to impaired gastrointestinal homeostasis and disease progression [1,5].

Several strategies have been developed to modulate the gut microbiome, with dietary management among the most effective and widely implemented approaches. In dogs, dietary interventions may include Biologically Appropriate Raw Food (BARF) diets, home-cooked meals, disease-specific extruded commercial kibble, and specialized therapeutic diets, such as allergen-elimination diets or hydrolyzed protein formulations [6,7]. Such interventions can influence microbiome composition and function by altering nutrient availability and substrates for microbial fermentation, potentially affecting metabolite production, gut barrier function, and immune signaling [8,9,10].

In addition to dietary approaches, microbiome-modulating strategies such as probiotics, prebiotics, synbiotics, and postbiotics have gained increasing attention for supporting gut health in companion animals. These interventions act through different mechanisms: probiotics introduce beneficial live microorganisms, prebiotics provide substrates that favor selected microbial populations, and synbiotics combine both approaches to enhance microbial survival and activity in the gastrointestinal tract. However, their clinical impact may vary considerably according to formulation, dose, disease context, and individual microbiota composition. In veterinary medicine, this variability is particularly relevant, as the available evidence remains limited and many commercial products still lack robust clinical validation [11,12,13,14,15].

Within this landscape, postbiotics are emerging as promising tools because they include inactivated microorganisms and/or bioactive microbial components with potential health benefits [16]. Among them, short-chain fatty acids, including acetic, propionic, and butyric acids and their salts, are especially relevant microbial-derived compounds associated with gut health. Butyrate, in particular, supports intestinal epithelial metabolism and barrier function. However, its direct administration may be limited by rapid absorption in the upper gastrointestinal tract and poor palatability [17,18].

Another emerging approach for modulating the gut microbiome involves the use of functional botanical ingredients, particularly those derived from Medicinal and Aromatic Plants (MAPs). These plant ingredients and plant-derived bioactive compounds are increasingly being framed within the category of phytobiotics, which refers to natural plant-based substances or extracts used to promote host health, support intestinal function, and modulate the microbiome [19,20].

MAPs are rich sources of bioactive secondary metabolites, including polyphenols, alkaloids, flavonoids, glycosides, terpenes, and essential oils [21]. These compounds have been associated with antioxidant, anti-inflammatory, and antimicrobial activities and may also influence gut microbial composition and activity and serve as substrates for microbial biotransformation [22].

Among MAPs, *Aloe vera*, *Artemisia annua*, and *Curcuma longa* have gained increasing attention due to their phytochemical profiles and reported biological activities, including antioxidant, anti-inflammatory, antimicrobial, and other health-related effects [23,24,25].

*Aloe vera* has been associated with anti-inflammatory, antimicrobial, detoxifying, and wound-healing effects [24]. Its polysaccharides have been reported to modulate gut microbiota composition by reducing pathogenic bacteria such as *Escherichia coli* and increasing beneficial genera, including *Lactobacillus* spp. and *Bifidobacterium* spp. [26]. *Aloe vera* gel may also support intestinal barrier integrity and microbial balance through effects on tight junction function, intestinal permeability, and serotonin-related pathways [27,28]. In dogs, *Aloe vera* is included in some supplements and functional products, although dog-specific clinical evidence regarding intestinal benefits remains limited [29,30,31,32]. From a safety perspective, purified *Aloe vera* gel should be distinguished from whole-leaf extracts, as aloin, an anthraquinone toxic to dogs, is mainly found in the outer leaf layer and is typically removed during processing.

*Artemisia annua* is widely recognized as the natural source of artemisinin, its main bioactive compound, which is extensively used in malaria treatment due to its potent antiparasitic activity [33]. The plant also contains flavonoids, phenolic acids, and polysaccharides associated with anti-inflammatory, antioxidant, and antimicrobial properties [25,34]. In relation to intestinal health, *Artemisia annua* has been reported to reduce pro-inflammatory cytokines [35], modulate immune-related markers [34], and increase gut microbiota diversity while promoting beneficial bacterial populations and reducing potentially pathogenic microorganisms [36,37]. Although evidence regarding its intestinal benefits in dogs remains limited, veterinary-relevant studies have reported antiparasitic and anticancer properties, together with evidence supporting its safety in animal applications [25,38].

*Curcuma longa*, commonly known as turmeric, is particularly valued for curcumin, its principal bioactive compound, which has demonstrated antioxidant, anti-inflammatory, antimicrobial, and gut-modulatory activities [23,39,40,41]. In dogs, dietary curcumin or *Curcuma longa* supplementation has been associated with improvements in antioxidant capacity and modulation of hematological and inflammatory markers [42]. Additional studies have reported downregulation of pro-inflammatory genes and changes in plasma markers consistent with reduced systemic inflammation and improved antioxidant and immune status [43].

Despite the available evidence on the individual effects of these plants on animal health, no studies have evaluated their combined use in dogs. Therefore, the objective of this study was to assess the effects of combined supplementation with *Aloe vera*, *Artemisia annua*, and *Curcuma longa* as a strategy to support intestinal health and modulate the gut microbiome in dogs.

## 2. Materials and Methods

### 2.1. Animals and Study Population

A total of 18 healthy dogs were recruited for the study through pet owners, who voluntarily enrolled their dogs. Eligible participants were selected based on predefined inclusion and exclusion criteria. A structured questionnaire was administered to collect information on both dog owners and participating animals, including age, body weight, breed, sex, medical history, medication use, diet, defecation patterns, physical activity, stress levels, neuter status, and deworming status. A summary of the main characteristics and diets of the animals included in this study is provided in Supplementary Table S1. As this was designed as a pilot exploratory study, the sample size was determined based on the feasibility of recruiting healthy dogs that met the inclusion criteria and completing the 30-day intervention and sampling protocol.

Only clinically healthy dogs without diagnosed diseases and not receiving medications or dietary supplements that could interfere with the study outcomes were included. Dogs that did not meet these criteria, or that had not been dewormed, were excluded. No underlying medical conditions requiring treatment were identified in any enrolled animal. Written informed consent was obtained from all owners prior to participation.

Regarding diet, 42.85% of the dogs were fed dry food as their primary diet, while 33% received a combination of dry and wet food. The remaining animals followed alternative dietary regimens, including raw and natural food-based diets. Dietary patterns were maintained throughout the study period to minimize potential confounding effects on the fecal microbiome.

### 2.2. Study Design and Experimental Groups Design

A parallel, placebo-controlled pilot study was conducted, in which dog owners were unaware of the treatment assigned to their dogs. No formal randomization was performed; instead, animals were allocated to two groups balanced by sex, age, body weight, and neuter status: (1) a Control group supplemented with microcrystalline cellulose as a placebo (*n* = 9); and (2) an Experimental group receiving a combination of *Aloe vera*, *Artemisia annua*, and *Curcuma longa* (*n* = 9).

### 2.3. Supplementation Protocol

Dogs in the Control group received 100 mg/10 kg of microcrystalline cellulose. Dogs in the Experimental group received a total dose of 775 mg/10 kg, consisting of 25 mg/10 kg of *Aloe vera*, 200 mg/10 kg of *Artemisia annua*, 500 mg/10 kg of *Curcuma longa*, and 50 mg/10 kg of microcrystalline cellulose. All three plants were administered as compressed powder in capsule form. The relative proportions of the botanical ingredients were established taking into account published evidence on their use in dogs, together with dosage ranges reported for canine formulations and commercially available products.

Each capsule contained up to 350 mg of plant material, and dogs received the number of capsules corresponding to their body weight and assigned group. When only one capsule per day was required, it was administered in the morning, preferably before breakfast. When two or more capsules were required, the daily dose was divided into two administrations, in the morning and evening. Caregivers were instructed not to modify the recommended dose and, in the event of a missed dose, not to double the subsequent administration, in order to avoid exceeding the recommended dosage.

### 2.4. Plant Material

The plant materials used in the experimental formulation included *Aloe vera* extract 200:1, standardized *Artemisia annua* containing 2.6 mg of artemisinin per capsule, and *Curcuma longa* containing 10 mg of curcumin per capsule. *Artemisia annua* was produced at the Biotech Tricopharming Research facilities in Tenerife, Spain. *Aloe vera* and *Curcuma longa* were provided by Nako Naturals GmbH (Bi de Forstkoppeln 6a, 21449 Radbruch, Germany) and Corexnat (C/Las Guirnaldas, Polígono los Puertas 29792 Cajiz, Málaga, Spain), respectively.

### 2.5. Fecal Sample Collection

After owners signed the informed consent form, study kits were shipped for fecal sample collection using DNA/RNA Shield Fecal Collection Tubes (Zymo Research, Irvine, CA, USA), which ensure nucleic acid stabilization at the point of collection. The treatment period lasted 30 days, with fecal samples collected on Day 0 and Day 30.

### 2.6. DNA Extraction and Shotgun Metagenomic Sequencing

Once all samples had been collected, they were sent to BioLab (Ascoli Piceno, Italy) for DNA extraction. DNA was standardly isolated using QIAamp DNA Microbiome Kit (Qiagen, Hilden, Germany) according to the manufacturer’s instructions. The extracted DNA was then forwarded to IGA Technology (Udine, Italy) for library preparation and whole-metagenome sequencing.

The Ovation^®^ Ultralow V2 DNA-Seq Library Preparation Kit (Tecan Genomics, Redwood City, CA, USA) was used for library preparation according to the manufacturer’s instructions. Both the input DNA and final libraries were quantified by Qubit 2.0 Fluorometer (Invitrogen, Carlsbad, CA, USA) and quality tested by Agilent 2100 Bioanalyzer High Sensitivity DNA assay (Agilent technologies, Santa Clara, CA, USA). Libraries were then prepared for sequencing and sequenced on NovaSeq 6000 (Illumina, San Diego, CA, USA) in paired-end 150 bp mode.

### 2.7. Alpha and Beta Diversity

Alpha diversity refers to the diversity of microbial taxa within a given sample. It combines two main components: richness, which reflects the number of different taxa identified, and evenness, which describes how evenly individuals are distributed among those taxa. To quantify these aspects, alpha diversity was evaluated using several indices, Chao1 and Observed-richness (for species richness); and Shannon, Simpson, and Fisher (for overall diversity and evenness). Outliers were removed (*p* < 0.05) to ensure robust comparisons across samples. Alpha diversity indices were calculated for each sample and subsequently compared statistically between groups.

In contrast, beta diversity describes differences in microbial community composition between samples or groups. In this study, beta diversity was evaluated to compare the Control and Experimental groups at Day 30 using the Bray–Curtis dissimilarity metric, where values range from 0 (identical community composition) to 1 (completely different community composition).

### 2.8. Bioinformatic and Statistical Analysis

FastQC software v0.11.4 was used to assess the quality of the raw sequencing data. Adapter sequences and low-quality reads (Phred score ≤ 25) were removed using BBDuk v35.85. High-quality sequences were then mapped against the dog reference genome (GCA_000002285.4) using minimap2 v2.25 (r1173). Unmapped reads were extracted with SAMtools 1.17 and subsequently analyzed with the GAIA pipeline v2.1.0 [44] for taxonomic classification based on the NCBI database. Functional annotation of the microbiome was performed with the SUPER-FOCUS pipeline v0.34 [45] using default parameters.

Once the read count tables for each taxonomic classification had been obtained, they were processed in R Studio 2026.05.1+225 (R Core Team, 2020) using the packages phyloseq, vegan, and DESeq2 to calculate rarefaction curves, relative abundance using the centered log-ratio (CLR) method, alpha diversity indices, and differential abundance analysis. For the latter, an adjusted *p*-value (*p*.adj) threshold of 0.05 was applied, and only species with a minimum of 10 reads across all samples were considered.

Differential abundance was performed with DESeq2 using design ~ condition, contrasting Experimental vs. Control at Day 30 only (unpaired). Counts were normalized with DESeq2 median-of-ratios size factors.

Statistical analyses and data visualization were performed in R Studio 2026.05.1+225 at the Barcelona office of Biotech Tricopharming Research (Barcelona, Spain). Graphs were generated using the ggplot2 package v4.0.3 in R version 4.5.2. Significant differences in alpha diversity were assessed using Wilcoxon rank-sum tests. Differential abundance of taxa and functions was assessed using DESeq2 with Benjamini–Hochberg FDR correction.

## 3. Results

### 3.1. Metagenomic Sequencing Metrics and Quality Control

Prior to downstream analyses, the raw sequencing data were quality-checked to remove low-quality regions while retaining the longest high-quality portions of each read. A minimum read length of 35 bp and a minimum quality score of 25 were applied to enhance the reliability of the dataset.

On average, 18.5 million reads per sample were produced. The number of reads per sample before and after trimming, as well as the number of reads assigned at the taxonomic level, is summarized in Supplementary Table S2. Host-derived reads were removed, and only reads assigned to genera with at least two counts in at least two samples were retained to reduce potential false positives.

### 3.2. Taxonomic Composition of the Canine Gut Microbiome

Taxonomic profiling was conducted using GAIA (version 2.1.0, Sequentia Biotech, Barcelona, Spain). In this workflow, paired-end reads were aligned against a curated reference database, the best alignments were extracted, and a Lowest Common Ancestor (LCA) algorithm was applied. Identity and coverage thresholds were then enforced, and the taxonomic assignments were summarized.

**Figure 1:**
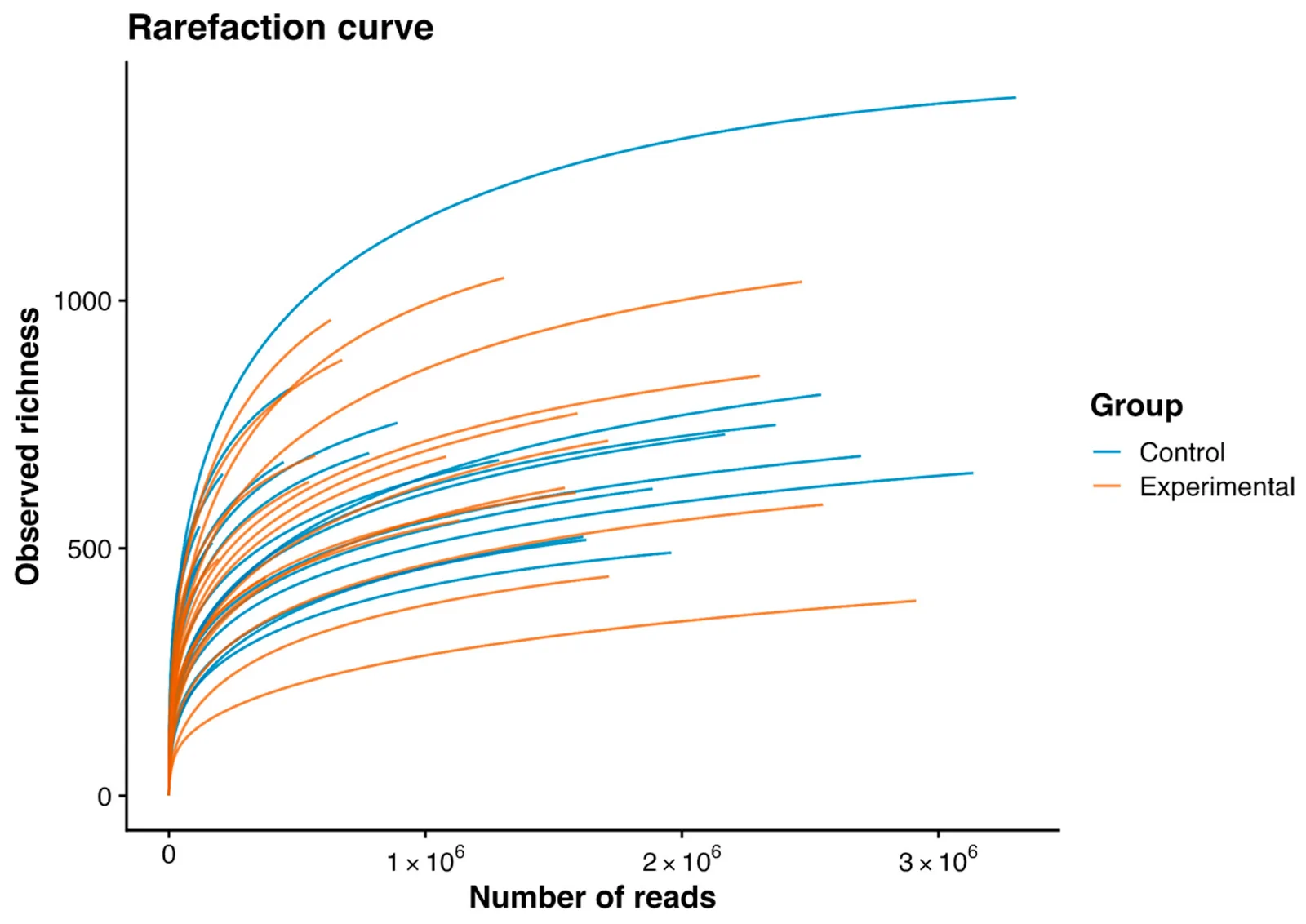
Rarefaction curve. The number of reads is shown on the *x*-axis and the alpha-diversity richness is shown on the *y*-axis.

To assess whether sequencing depth was sufficient to capture microbial diversity, rarefaction curves were generated (Figure 1). These plots, showing the number of reads on the *x*-axis and alpha-diversity richness on the *y*-axis, illustrate how species richness increases with sequencing effort. Steep initial slopes indicate the detection of many new taxa, with additional reads, whereas flattened slopes suggest that most of them had already been captured. Convergence of the rarefaction curve toward a horizontal asymptote indicates that sequencing depth was sufficient to represent the microbial communities accurately.

**Figure 2:**
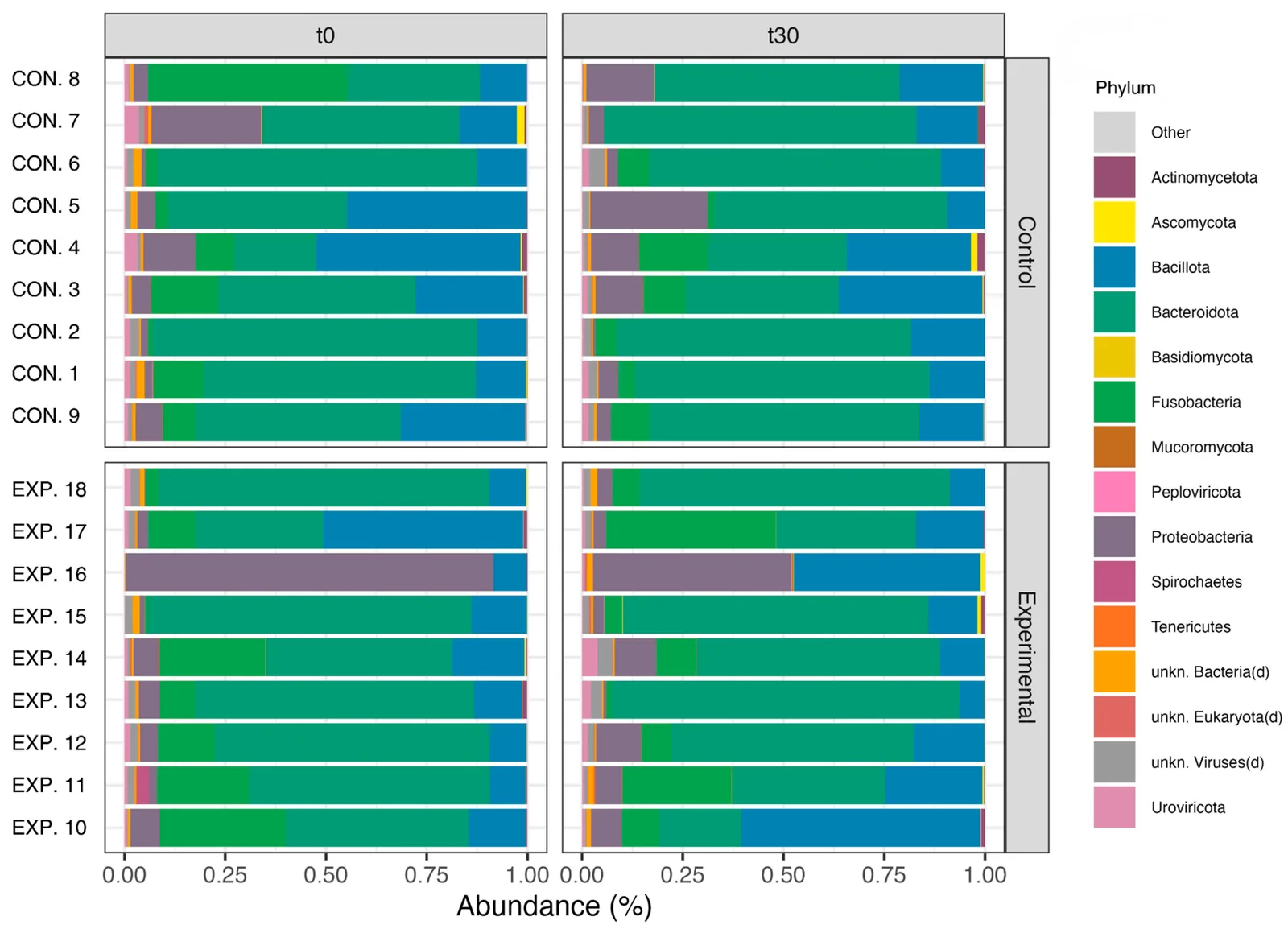
Taxonomic profiling of the microbiome at phylum level. The 15 taxa with the highest mean counts across all samples are shown, while the remaining taxa are grouped as ‘Other’. Each row represents one dog, and dogs are grouped vertically by treatment group (Control, Experimental).

**Figure 3:**
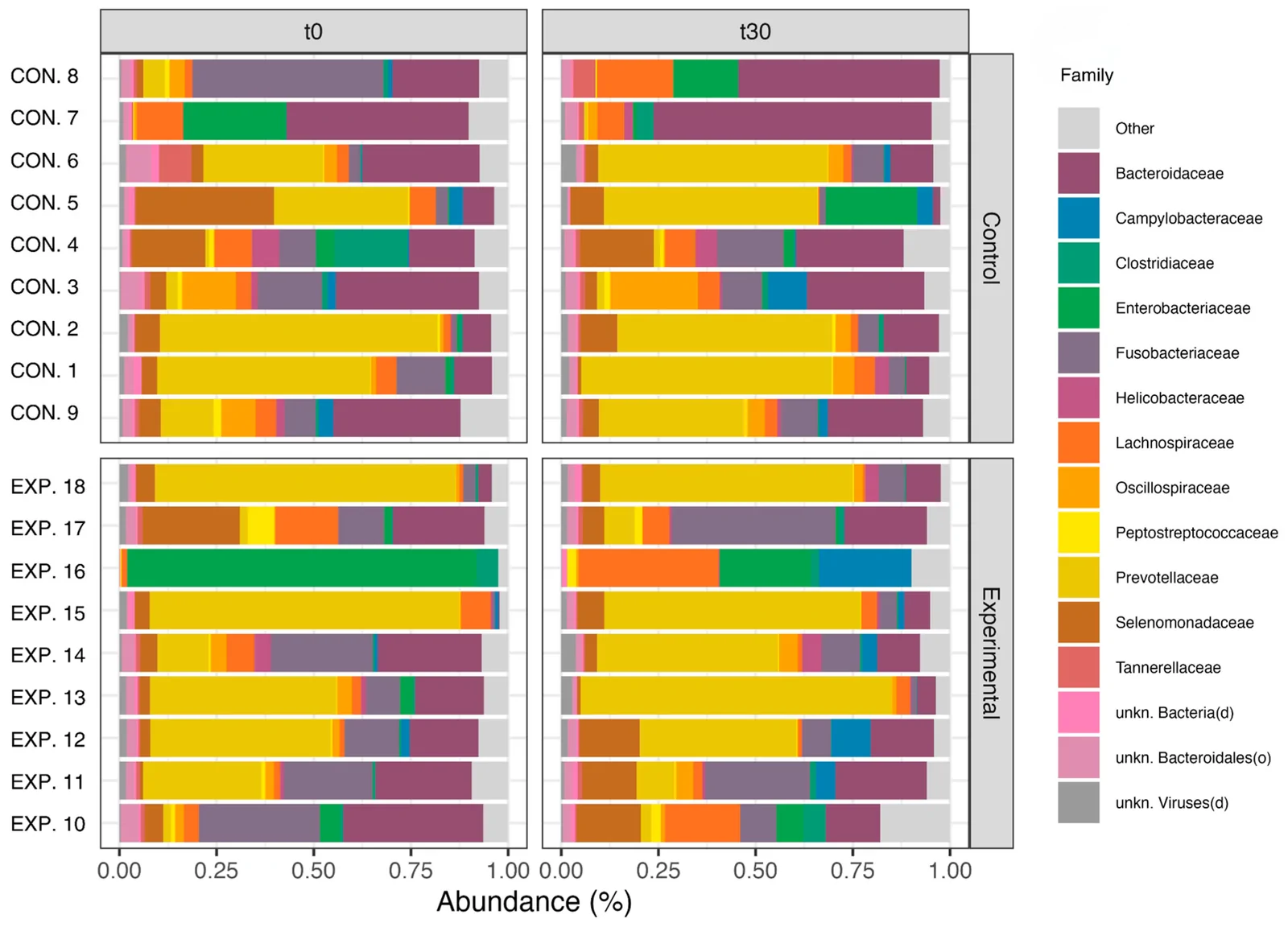
Taxonomic profiling of the microbiome at family level. The 15 taxa with the highest mean counts across all samples are shown, while the remaining taxa are grouped as ‘Other’. Each row represents one dog, and dogs are grouped vertically by treatment group (Control, Experimental).

**Figure 4:**
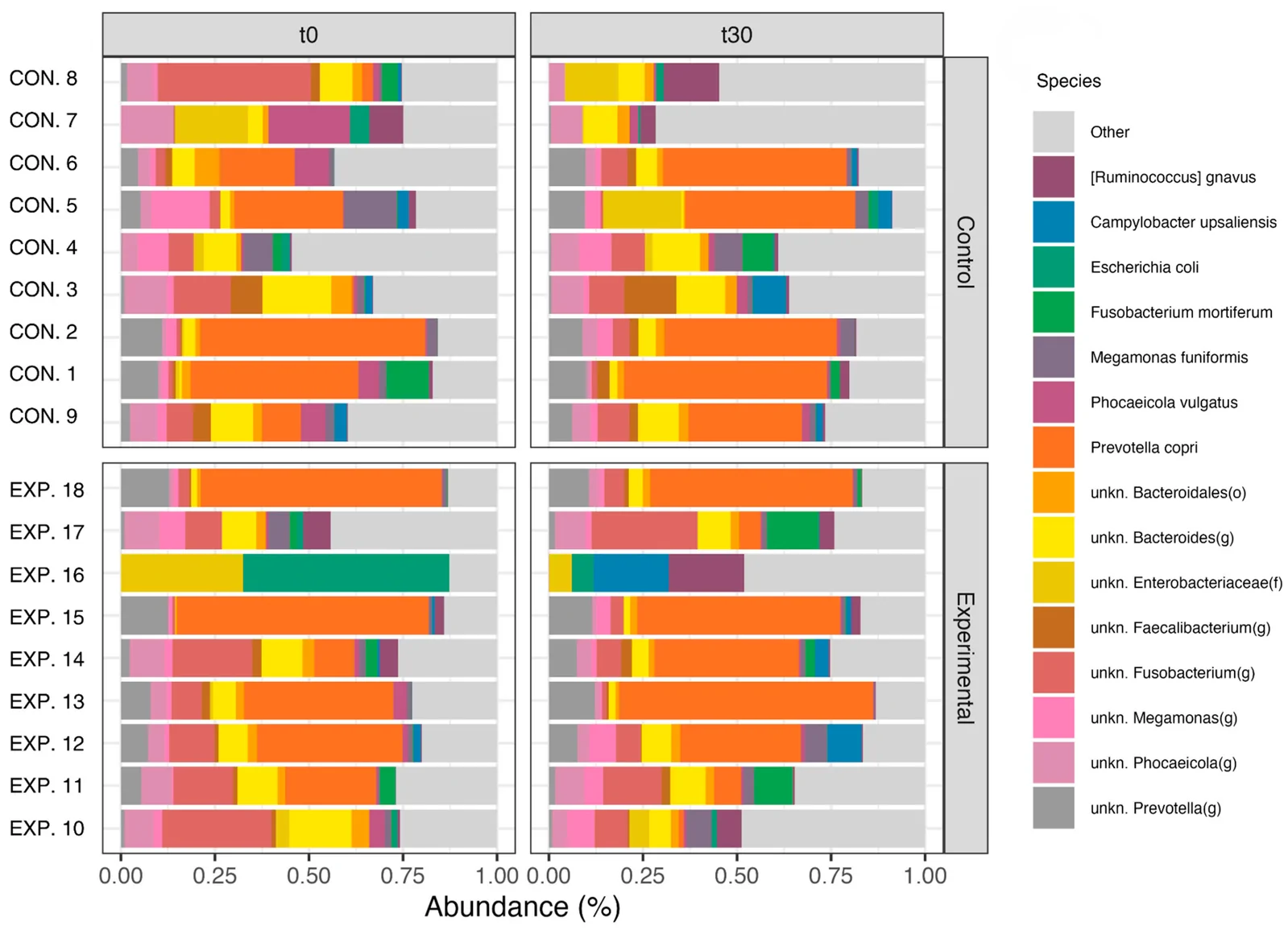
Taxonomic profiling of the microbiome at species level. The 15 taxa with the highest mean counts across all samples are shown, while the remaining taxa are grouped as ‘Other’. Each row represents one dog, and dogs are grouped vertically by treatment group (Control, Experimental).

The trimmed reads were further processed for taxonomic classification and assigned at the phylum (Figure 2), family (Figure 3), and species (Figure 4). The 15 taxa with the highest mean counts across all samples are shown, while the remaining taxa are grouped as “Other”.

At the phylum level, the taxonomic composition was highly consistent across all samples. Bacteroidota was the most abundant phylum in every sample, representing more than 50% of the relative abundance, while Bacillota and Fusobacteriota were the second most prevalent phyla, with their relative contributions varying slightly between samples. Overall, the relative abundance patterns were similar across individual dogs, as illustrated in Figure 2, highlighting the stability of the microbial community structure within the cohort.

This phylum-level profile is consistent with the typical gut microbiome composition observed in healthy dogs, in which Bacillota (formerly Firmicutes), Bacteroidota (formerly Bacteroidetes), Fusobacteriota, Proteobacteria, and Actinobacteriota are the dominant phyla [1].

At the family level, *Bacteroidaceae*, *Prevotellaceae*, and *Fusobacteriaceae* were the most abundant taxa across all samples, collectively representing the majority of the microbial community. No relevant differences in the relative abundance of the dominant families were observed between groups.

At the species level (Figure 4), the overall community structure appears relatively stable between Day 0 and Day 30 in both the Control and Experimental groups. *Prevotella copri*, *Fusobacterium mortiferum*, and *Phocaeicola vulgatus* remained among the dominant taxa across samples. These species are commonly reported as commensal members of the canine gut microbiome and are linked with carbohydrate and protein metabolism, contributing to overall gut microbial homeostasis [1,46].

By Day 30, no major compositional shifts were observed at the group level.

Interestingly, at the individual-level, one animal in the Experimental group (EXP. 16) displayed a high abundance of *Escherichia coli* at Day 0, which markedly decreased by Day 30 (from 54.8% abundance to 5.8%). This reduction may be associated with supplementation with the three plants, potentially supporting the modulation of opportunistic bacteria.

### 3.3. Effects on Microbial Diversity and Composition

The analysis of microbiome composition provides valuable insights into the role of microorganisms in canine health. Evaluating the intrinsic diversity within each sample offers a relative snapshot of the host’s intestinal health status.

Alpha diversity, which describes the diversity of species within a single community or sample, considering features such as richness (species number) and evenness (abundance balance), were calculated in relation to age and body weight (Supplementary Figure S1). Alpha diversity was evaluated using several indices, Chao1 and Observed-richness (for species richness); and Shannon, Simpson, and Fisher (for overall diversity and evenness). No statistically significant differences were observed for either variable. However, animals in the 1–10 kg group tended to show lower Shannon diversity than heavier animals, suggesting a possible, although non-significant, increase in diversity with body weight.

Regarding age, a non-significant progressive decrease in evenness was observed with increasing age. Although this trend did not reach statistical significance, it is consistent with previous reports suggesting reduced microbiome stability and increased inter-individual variability in older animals, potentially associated with age associated physiological changes [47].

**Figure 5:**
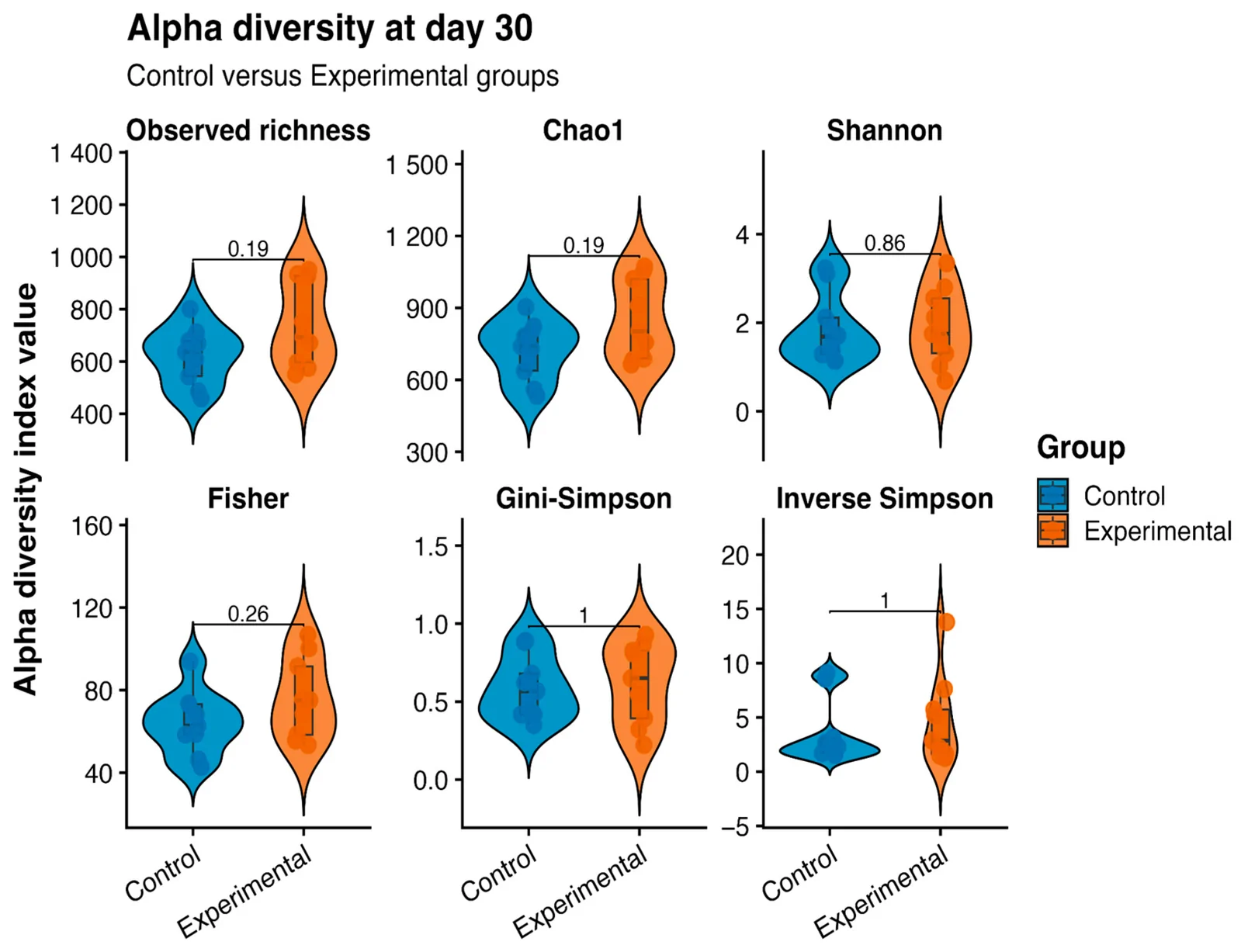
Alpha-diversity indices at Day 30 in the Control and Experimental groups. Violin plots with embedded boxplots show the distribution of each alpha-diversity metric in the Control (blue) with Experimental (orange) groups. Statistical significance between groups was assessed using the Wilcoxon rank-sum test.

This analysis was applied to both the Control and Experimental groups at Day 30 to evaluate potential changes in microbial community complexity in response to treatment. No statistically significant differences were detected between the groups (Figure 5), indicating comparable microbial diversity. However, numerically, median observed richness, Chao1, and Fisher’s alpha were higher in the Experimental group than in the Control group at Day 30 (observed richness: 1132 vs. 859, *p* = 0.19; Chao1: 1619 vs. 1281, *p* = 0.19; Fisher’s alpha: 142 vs. 104, *p* = 0.26), suggesting a possible trend toward greater microbial richness following treatment.

**Figure 6:**
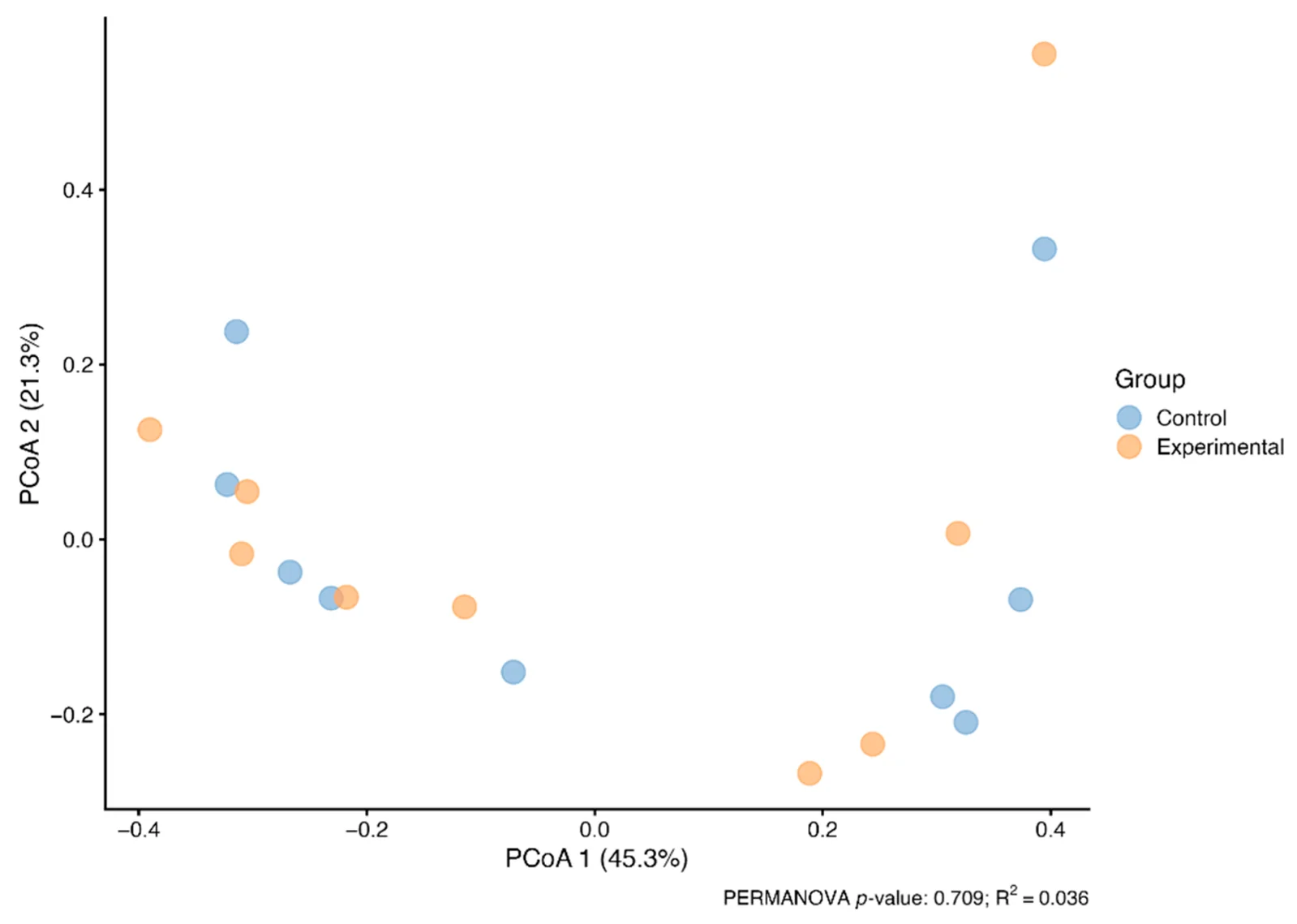
Principal coordinate analysis (PCoA) based on Bray–Curtis dissimilarities at Day 30. Samples are colored according to group (Control and Experimental). PCoA1 and PCoA2 explain 45.3% and 21.3% of the variation, respectively. Differences in community composition between groups were assessed using PERMANOVA (R^2^ = 0.036, *p* = 0.709).

Complementary to this, beta diversity, which describes the differences in species composition between communities or samples, was evaluated to assess differences in microbial composition between samples. Principal Coordinates Analysis (PCoA) based on Bray–Curtis dissimilarities showed substantial overlap between the Control and Experimental groups at Day 30, with no clear group-specific clustering, suggesting that overall microbial community composition was largely similar at Day 30 (Figure 6). This observation was supported by PERMANOVA, which indicated no significant differences in community composition between groups (R^2^ = 0.036, *p* = 0.709).

### 3.4. Differentially Abundant Taxa Between Groups

**Figure 7:**
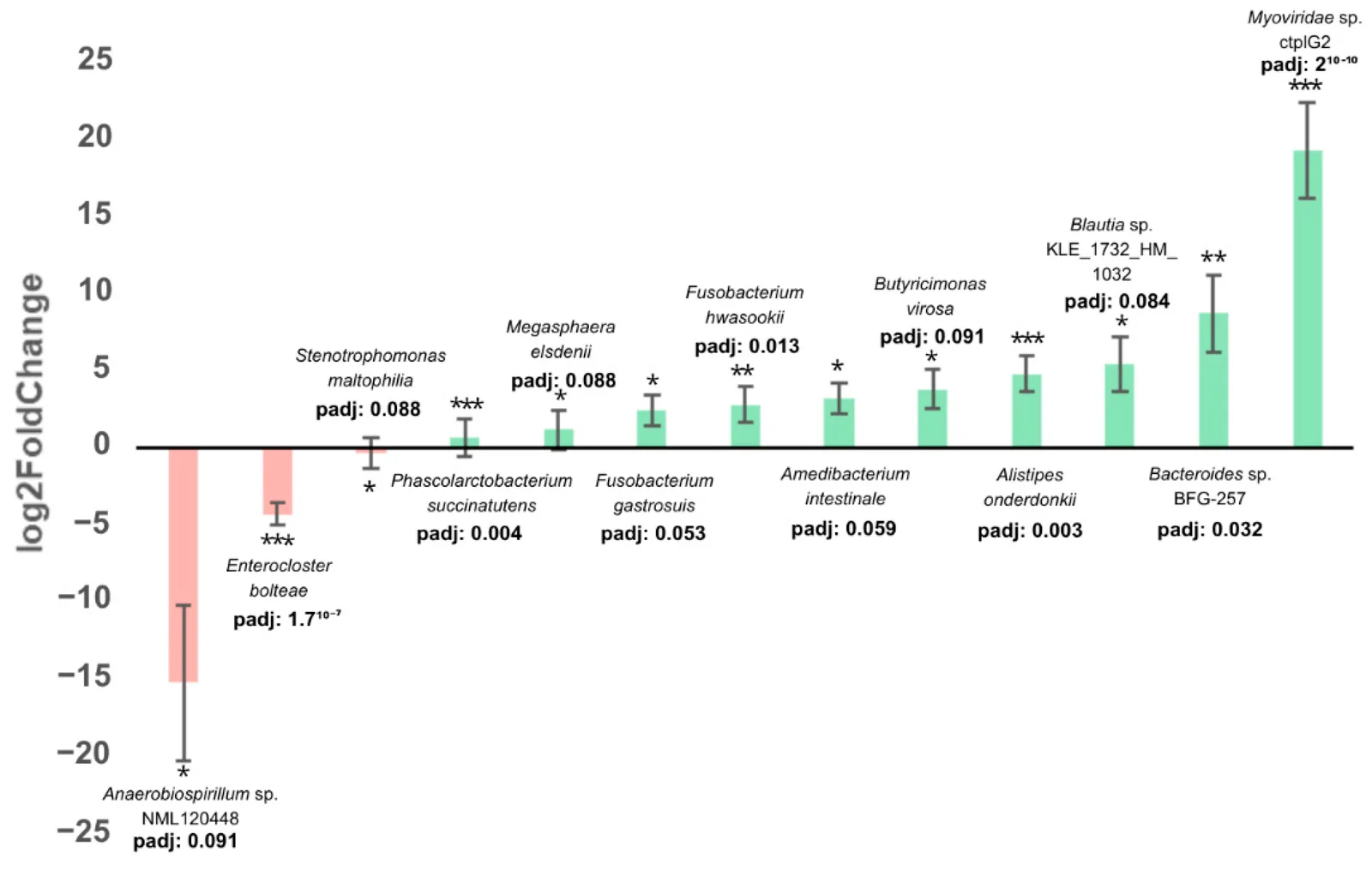
Differentially abundant microorganisms between the Control and Experimental groups at Day 30. The bar plot shows the log2 fold change for each microorganism, calculated as Experimental relative to Control. Positive log2 fold change values (green) indicate higher abundance in the Experimental group, whereas negative log2 fold change values indicate higher abundance in the Control group. Error bars represent the standard error, and asterisks indicate statistical significance: *p* < 0.1 (*), *p* < 0.05 (**), and *p* < 0.01 (***).

Differential abundance analysis was performed to identify microbial species that differed significantly between the Control and Experimental groups at Day 30. The main representative microorganisms showing significant differences in relative abundance between groups (FDR < 0.1) are presented in Figure 7. The complete raw data are provided in Supplementary Table S3.

Notably, several bacteria taxa associated with gut metabolic activity and short-chain fatty acid (SCFA) production were significantly increased in the Experimental group, including *Megasphaera elsdenii*, *Fusobacterium* spp., *Butyricimonas virosa*, *Blautia* spp., *Phascolarctobacterium succinatutens*, and *Bacteroides* spp. These taxa are known to contribute directly or indirectly to microbial fermentation pathways involved in the production of key SCFAs, including butyrate, propionate, and acetate [48].

In particular, butyrate-associated genera such as *Blautia* and *Butyricimonas* were enriched in the Experimental group. *Bacteroides* spp. were also increased, consistent with their known role in the degradation of complex polysaccharides and the generation of metabolically beneficial end products [49]. In addition, *Phascolarctobacterium succinatutens*, involved in propionate production, was more abundant in the Experimental group [50]. The supplemented group also showed increased abundance of *Amedibacterium intestinale* and *Alistipes onderdonkii*, two recently gut-associated taxa that have been reported in both humans and dogs [51,52,53,54,55].

In contrast, *Enterocloster bolteae* and *Anaerobiospirillum* spp., were significantly reduced in the Experimental group. These taxa have previously been associated with dysbiosis or gastrointestinal disturbances in certain contexts [1,56].

*Stenotrophomonas maltophilia* was also reduced in the Experimental group. This species is an opportunistic, multidrug-resistant, Gram-negative bacterium that has been reported in dogs, including in association with urinary tract infections and chronic respiratory disease [57,58].

Overall, these results suggest a possible shift in the Experimental group toward a microbial profile characterized by a higher abundance of taxa previously associated with SCFA production and a lower abundance of taxa that have been linked to opportunistic behavior, pro-inflammatory contexts, or dysbiotic states. Among viral taxa, *Myoviridae* spp. bacteriophages also showed a marked increase in relative abundance in the Experimental group. This shift occurred alongside the reduction in several potentially opportunistic bacterial taxa. However, further phage-host interaction analyses would be required to determine whether these viral shifts directly contributed to bacterial community changes.

### 3.5. Functional Profiling and Potential Scfa-Related Pathways

To complement the taxonomic analysis, metagenomic functional profiling was performed using SUPER-FOCUS. This approach was used to characterize the functional potential of the fecal microbial communities and to identify gene functions and metabolic pathways that were differentially represented between the Control and Experimental group at Day 30.

Differential abundance analysis was then performed to identify functional pathways that differed between the Control and Experimental groups at Day 30. Given the exploratory nature of the study and the aim of providing a broader overview of potentially relevant functional changes, an adjusted *p*-value threshold of <0.1 was applied. The main differentially represented functions meeting this threshold are presented in Table 1, while the complete dataset is provided in Supplementary Table S4.

Overall, several functional pathways were differentially represented between groups, with both decreased and increased relative abundance observed in the Experimental group. Reduced pathways included those related to carbohydrate utilization and sugar transport, such as the unknown carbohydrate utilization cluster Ydj and the cluster Ytf/putative sugar transporter, as well as pathways related to the glyoxylate bypass, aromatic amino acid catabolism, intracellular septation in *Enterobacteria*, capsular surface virulence antigen loci, and iron siderophore sensor and receptor systems. In contrast, pathways related to GABA and putrescine metabolism, serine endopeptidase activity, and additional predicted functions of unknown or poorly characterized biological relevance were increased in the Experimental group.

Together, these findings indicate distinct differences in the predicted functional profile of the fecal microbiome between the Control and Experimental groups at Day 30.

## 4. Discussion

The fecal microbiome is increasingly recognized as a non-invasive indicator of gastrointestinal health and as a potential marker of extraintestinal disorders. Changes in fecal microbial composition and function have been associated not only with gastrointestinal conditions, such as dysbiosis, chronic enteropathies, and intestinal inflammation, but also with systemic processes linked to gut-organ axes, including immune, metabolic, dermatological, and neurological alterations [59,60,61]. Although fecal samples do not fully represent all intestinal niches, their easy collection makes them a practical biomarker for monitoring digestive status, identifying microbial imbalances, and evaluating responses to nutritional or therapeutic interventions [1,56,62].

Several approaches have been employed to characterize the canine gut microbiome. Traditional techniques such as microbial culture and fluorescence in situ hybridization (FISH) provided early insights but are limited by cultivability constraints and low taxonomic resolution [63]. The advent of next-generation sequencing (NGS) has transformed the field, enabling massively parallel sequencing of microbial DNA and RNA [64]. Within these methods, 16S rRNA gene sequencing is widely used but generally restricted to genus-level classification, whereas shotgun metagenomics provides higher taxonomic resolution, functional insights, and inclusion of non-bacterial microbes, as well as comprehensive assessment of microbial diversity despite higher costs and technical demands [65]. This approach is especially valuable in canine research, as the gut microbiome of dogs remains less extensively characterized than that of humans or other model species [4].

Based on the role of the intestinal microbiome as an indicator of gut health, together with the growing interest in alternatives to probiotics, prebiotics, postbiotics, and synbiotics, and the increasing use of botanicals within the emerging concept of phytobiotics, this study aimed to evaluate the effects of supplementing healthy dogs with a combination of three well-known MAPs (*Aloe vera*, *Artemisia annua*, and *Curcuma longa*). Although the individual effects of these MAPs on intestinal health have been evaluated in several species, and their benefits have been widely reported in non-canine models, evidence in dogs remains limited. Moreover, the impact of their combined supplementation on the canine intestinal microbiome has not previously been characterized.

Therefore, this study assessed microbiome changes after 30 days of supplementation with a combination of *Aloe vera*, *Artemisia annua*, and *Curcuma longa* in the Experimental group compared with the Control group. Microbiome composition was evaluated by comparing Day 30 samples from the Experimental group with those from the Control group.

No significant differences in alpha diversity were observed between the Experimental and Control groups after 30 days of supplementation. This may be partly explained by the high inter-individual variability of the canine gut microbiome. Individual microbial profiles can be influenced by factors such as diet, age, breed, environment, host physiology, and baseline microbiome composition. In this study, healthy dogs were included, and diet type was balanced between the Control and Experimental groups. However, the wide age range and breed diversity, intended to better represent the canine population, may have increased heterogeneity and limited the detection of group-level differences, particularly given the sample size.

Nevertheless, the supplemented group showed a slight, non-significant increase in richness-related indices, including observed richness, Chao1, and Fisher’s alpha, suggesting a modest expansion of low-abundance taxa rather than a broad shift in evenness. Thus, the absence of major changes in alpha-diversity may indicate microbiome stability, while the observed richness trend suggests possible selective modulation. This should be confirmed in larger longitudinal studies comparing pre- and post-supplementation samples from the same dogs. This interpretation is consistent with previous canine studies using prebiotic-, probiotic-, synbiotic-, or postbiotic-based strategies, in which supplementation did not always result in clear differences in alpha diversity but was associated with selective shifts in bacterial taxa, short-chain fatty acid production, immune markers, or digestive health outcomes [66,67].

Differential abundance analysis suggested that supplementation was associated with selective microbial shifts rather than broad community-level restructuring. Several taxa with potential metabolic relevance were significantly enriched in the Experimental group, including *Megasphaera elsdenii*, *Fusobacterium* spp., *Butyricimonas virosa*, *Blautia* spp., *Phascolarctobacterium succinatutens*, and *Bacteroides* spp. Interestingly, all these taxa have been linked to microbial fermentation and short-chain fatty acid (SCFA)-related pathways, including acetate, propionate, and butyrate metabolism. SCFAs play important roles in gut health by supporting epithelial energy metabolism, intestinal barrier integrity, immune regulation, and host metabolic signaling [1,68,69,70,71,72]. Therefore, these taxonomic changes may reflect an enhanced fermentative potential and associated changes in SCFAs production; however, further studies, including direct metabolite quantification, are needed to confirm this interpretation.

Among the enriched taxa, *Phascolarctobacterium succinatutens* is of particular interest due to its ability to utilize succinate and potentially contribute to propionate production. Its increased abundance may therefore suggest enhanced succinate conversion capacity, which could be relevant because impaired succinate utilization has been associated with dysbiotic states in some gastrointestinal contexts [50,73]. In dogs, reduced abundance of *Phascolarctobacterium* spp. abundance has been reported in chronic enteropathy and small-cell lymphoma, suggesting that this genus may be associated with intestinal microbial balance [74]. Associations between *Phascolarctobacterium* and mood- or stress-related responses have also been reported, although this evidence is derived mainly from exploratory human studies [75,76]. Therefore, the increase observed in the present study may support future research on microbiome-metabolite interactions and gut–brain axis signaling, but it should not be interpreted as evidence of behavioral or neurological effects.

Two additional taxa, *Amedibacterium intestinale* and *Alistipes onderdonkii*, were also enriched in the Experimental group. *A. intestinale* is a recently described gut-associated bacterium with limited characterization in dogs, although available phenotypic data suggest that it may ferment carbohydrates and potentially produce SCFAs [51]. In humans, changes in *A. intestinale* abundance have been reported in disease-associated gut microbiome profiles, including pulmonary arterial hypertension; however, the biological significance of this taxon appears to be context-dependent [77].

*A. onderdonkii* has been reported in both humans and dogs, but its role in canine gastrointestinal health remains unclear. More broadly, associations involving the genus *Alistipes* in humans appear to be highly context-dependent, ranging from potentially protective to disease-associated patterns [52,53,54,55,78]. Thus, increases in *A. intestinale* and *A. onderdonkii* may reflect modulation of anaerobic gut-associated taxa after supplementation, but their biological significance in dogs remains uncertain and requires further investigation.

In parallel with the enrichment of fermentation-associated taxa, supplementation was also associated with a reduction in several bacterial taxa previously linked to opportunistic or dysbiosis-associated contexts. *Anaerobiospirillum* spp. showed a marked decrease in the Experimental group. These Gram-negative anaerobic rods can be part of the intestinal microbiota of dogs; however, it has also been reported as an opportunistic pathogen associated with peritonitis in dogs and with diarrhea or septicemia in immunocompromised humans [79,80,81,82,83]. Similarly, *Enterocloster bolteae*, another reduced species, is a spore-forming gastrointestinal bacterium whose role in dogs remains poorly characterized. In humans, however, *Enterocloster bolteae* has been associated with dysbiosis and several disease-related gut microbiome profiles, including autism spectrum disorder, multiple sclerosis, and inflammatory bowel disease [84,85]. Finally, *Stenotrophomonas maltophilia* was also reduced after supplementation. This species is an emerging opportunistic, multidrug-resistant Gram-negative bacterium that has been reported in dogs, where it has mainly been associated with urinary tract infections and chronic respiratory disease [57,58]. Overall, the reduction in these taxa may suggest a shift toward a microbial profile with a lower abundance of potentially opportunistic or dysbiosis-associated bacteria. However, given the size and exploratory nature of this pilot study, together with the fact that the study was conducted in clinically healthy dogs and fecal detection does not necessarily imply pathogenic activity, these findings should be interpreted cautiously. The observed reductions may represent a potentially favorable ecological shift, but confirmation in larger, adequately powered studies is required.

Among viral taxa, an increase in *Myoviridae*-like bacteriophages was observed in the Experimental group. Because many members of this group are lytic phages, this shift may reflect altered phage-bacteria dynamics within the gut ecosystem [86,87]. Increased lytic phage activity could contribute to bacterial turnover through density-dependent predation, thereby potentially limiting the expansion of certain bacterial populations and indirectly modifying competitive interactions among community members [88]. In addition, phage-mediated bacterial lysis can release intracellular nutrients and metabolites into the surrounding environment, potentially altering resource availability and influencing the growth of other community members [89,90]. Together, these ecological mechanisms could contribute to shifts in bacterial community structure and may be relevant to some of the bacterial taxa that decreased following supplementation. However, phage ecology is highly complex, and phage-host relationships were not directly characterized in the present study. Therefore, the observed increase in *Myoviridae*-like bacteriophages should be interpreted as an exploratory finding that may reflect restructuring of the microbial ecosystem, rather than as direct evidence of phage-mediated control of specific bacterial taxa.

Predicted functional profiling also suggested changes in microbial metabolic potential after supplementation with the three medicinal plants. Reduced representation of pathways related to intracellular septation in Enterobacteria, capsular surface virulence antigen loci, and iron acquisition systems was observed. These findings raise the hypothesis that supplementation may be associated with a lower representation of functions linked to bacterial proliferation and virulence-related traits. Since *Enterobacteriaceae* include several opportunistic and pathogenic members, the observed reductions may be consistent with a shift away from a more dysbiosis-associated functional profile [91,92]. However, this interpretation remains speculative and requires confirmation through targeted functional and experimental analyses.

Conversely, the enrichment of GABA metabolism pathways may suggest changes in microbial functions potentially involved in host-microbe communication and gut–brain axis signaling. Microbial GABA metabolism has been linked to intestinal homeostasis and neuroactive signaling pathways, although this interpretation remains exploratory in dogs [93,94]. Finally, the increase in serine endopeptidase-related pathways may reflect greater proteolytic potential within the microbial community, which could influence protein degradation, peptide turnover, and nutrient availability. Importantly, these functional findings should be interpreted cautiously because they reflect predicted metabolic potential rather than confirmed gene expression, metabolite production, enzymatic activity, or physiological effects. These observations therefore support the hypothesis of altered microbial functional potential, including reduced virulence-associated traits and possible changes in host-microbe communication capacity. However, additional metagenomic, transcriptomic, metabolomic, and enzymatic analyses would be required to determine whether these predicted pathways translate into measurable biological activity and to further elucidate their potential modes of action.

Overall, these results suggest that supplementation with *Aloe vera*, *Artemisia annua*, and *Curcuma longa* may promote selective and potentially favorable changes in the canine gut microbial ecosystem. However, several limitations should be considered. First, the relatively small sample size of this pilot study may have limited statistical power and reduced the ability to detect subtle group-level differences. Therefore, the findings should be considered preliminary and hypothesis-generating. In addition, individual variability in the canine gut microbiome may have influenced the magnitude and direction of the observed microbial changes, particularly given the naturally high inter-individual heterogeneity reported in dogs. This variability may partly explain the absence of significant differences in alpha diversity despite the detection of a slight increase in richness-related indices. Importantly, dietary heterogeneity could represent a relevant confounding factor in the present study. Although the distribution of diet types was balanced between groups, enrolled dogs consumed different types of diets, including raw food, wet or canned food, and dry food, each of which may exert distinct effects on gut microbial composition and function independently of botanical supplementation. This dietary heterogeneity, together with the wide age range and breed diversity, may also have influenced gut microbiome composition and contributed to inter-individual variability in the observed responses. Future studies including larger sample sizes, a more standardized dietary background, and a longitudinal design incorporating baseline (Day 0) and post-intervention (Day 30) comparisons within the same animals would help increase statistical power, reduce inter-individual variability, and enable a clearer assessment of the specific effects of phytobiotic supplementation.

Second, the intervention consisted of the simultaneous administration of three botanicals, which prevents the contribution of each individual ingredient from being disentangled. Therefore, it remains unclear whether the possible observed microbiota changes are attributable to one particular botanical or to the combined effect of *Aloe vera*, *Artemisia annua*, and *Curcuma longa*. However, the aim of the present study was not to determine potential synergistic or additive effects among the individual botanicals, but rather to evaluate whether their combined administration, as a single botanical formulation, could beneficially modulate the gut microbiome of healthy dogs. Nevertheless, future studies evaluating each botanical separately, would be valuable to clarify their individual effects on the canine gut microbiome.

Third, the interpretation of specific taxa remains limited by the lack of reference studies in dogs, particularly for recently described or poorly characterized microorganisms such as *Amedibacterium intestinale* and *Alistipes onderdonkii*. Lastly, because the findings are mainly based on compositional and predicted functional data, they cannot confirm changes in microbial activity, metabolite production, host physiological responses, SCFA concentrations, enzymatic activity, inflammatory markers, or clinical outcomes. Therefore, although these results require further validation, they provide novel, hypothesis-generating evidence that this botanical combination may influence the canine gut microbiome. Future studies incorporating targeted metabolomics (e.g., SCFA quantification), enzymatic assays, and longitudinal microbiome monitoring will be needed to determine whether the observed taxonomic and functional shifts translate into measurable biological effects.

In conclusion, although supplementation with *Aloe vera*, *Artemisia annua*, and *Curcuma longa* for 30 days did not induce statistically significant changes in fecal microbial diversity in healthy dogs; a trend toward modulation of the gut microbiota was observed. Additionally, the observed enrichment of taxa previously associated with fermentation processes and short-chain fatty acid production, together with the reduction in potentially opportunistic or dysbiosis-associated bacteria and apparent shifts in predicted functional pathways, may suggest a selective effect on specific components of the gut microbial ecosystem, potentially contributing to a more favorable microbial profile. Although these findings require confirmation, they provide preliminary evidence that this combination of botanical functional ingredients may represent a promising complementary strategy to support intestinal microbial balance in dogs.

## 5. Conclusions

This pilot study evaluated the effects of 30 days of supplementation with *Aloe vera*, *Artemisia annua*, and *Curcuma longa* on the intestinal microbiome of healthy dogs. Shotgun metagenomic sequencing was used to compare Day 30 fecal samples from plant-supplemented dogs with those from placebo-treated dogs, providing taxonomic and functional characterization of the microbial communities. These findings contribute to the growing interest in phytobiotics as nutritional tools for supporting gut health in dogs and provide a basis for further investigation. Although further studies integrating metabolomic, clinical, and functional analyses are needed, this pilot study provides preliminary evidence that this phytobiotic combination may represent a promising complementary strategy to support gut microbiome homeostasis in dogs.

## Figures and Tables

**Table 1:** Differentially represented functional pathways between the Control and Experimental groups at Day 30. Log fold change values represent changes in relative abundance in the Experimental group compared with the Control group. Negative values indicate a decreased relative abundance in the Experimental group, whereas positive values indicate an increased relative abundance. Only pathways with pFDR < 0.1 are shown.

Function	log2FoldChange	padj
Unknown carbohydrate utilization (cluster Ydj)	−2.895	0.013
Glyoxylate bypass	−2.607	0.036
Iron siderophore sensor & receptor system	−2.396	0.086
Cluster Ytf and putative sugar transporter	−2.197	0.039
Aromatic Amino Acid Catabolism	−1.054	0.013
Capsular surface virulence antigen loci	−0.625	0.013
Intracellular septation in Enterobacteria	−0.25	0.05
GABA and putrescine metabolism from clusters	0.189	0.013
CBSS-584.1.peg.841	0.293	0.013
EC 3.4.21.- Serine endopeptidase	0.446	0.02
CBSS-502800.3.peg.2785	0.731	0.082

## Data Availability

The original contributions presented in this study are included in the article/Supplementary Material. Further inquiries can be directed to the corresponding author.

## Supplementary Materials

The following supporting information can be downloaded at: https://www.mdpi.com/article/10.3390/vetsci13090877/s1, Table S1: Main characteristics of the animals included in the study, including age, body weight, breed, and diet; Table S2: Sequencing read-processing and quality control metrics; Table S3: Raw taxonomic abundance data at the species level; Table S4: Raw functional abundance data; Figure S1: Shannon alpha diversity across body weight and age groups, with adjusted *p*-values indicating significant differences at padj < 0.05.
